# Hypoxic Signaling and Cholesterol Lipotoxicity in Fatty Liver Disease Progression

**DOI:** 10.1155/2018/2548154

**Published:** 2018-05-31

**Authors:** Oren Tirosh

**Affiliations:** Institute of Biochemistry, Food Science and Nutrition, The R.H. Smith Faculty of Agriculture, Food and Environment, The Hebrew University of Jerusalem, Rehovot, Israel

## Abstract

Cholesterol is the only lipid whose absorption in the gastrointestinal tract is limited by gate-keeping transporters and efflux mechanisms, preventing its rapid absorption and accumulation in the liver and blood vessels. In this review, I explored the current data regarding cholesterol accumulation in liver cells and key mechanisms in cholesterol-induced fatty liver disease associated with the activation of deleterious hypoxic and nitric oxide signal transduction pathways. Although nonalcoholic fatty liver disease (NAFLD) affects both obese and nonobese individuals, the mechanism of NAFLD progression in lean individuals with healthy metabolism is puzzling. Lean NAFLD individuals exhibit normal metabolic responses, implying that liver damage is not associated with impaired metabolism per se and that direct lipotoxic effects are crucial for disease progression. Several redox and oxidant signaling pathways involving cholesterol are at play in fatty liver disease development. These include impairment of the mitochondrial and lysosomal function by cholesterol loading of the inner-cell membranes; formation of cholesterol crystals and hepatocyte degradation; and crown-like structures surrounding degrading hepatocytes, activating Kupffer cells, and evoking inflammation. The current review focuses on the induction of liver inflammation, fibrosis, and steatosis by free cholesterol via the hypoxia-inducible factor 1*α* (HIF-1*α*), a main oxygen-sensing transcription factor involved in all stages of NAFLD. Cholesterol loading in hepatocytes can result in chronic HIF-1*α* activity because of the decreased oxygen availability and excessive production of nitric oxide and mitochondrial reactive oxygen species.

## 1. Nonalcoholic Fatty Liver Disease (NAFLD) and Lipotoxicity

### 1.1. Pathology

The liver is a major site for the synthesis, oxidation, metabolism, storage, and distribution of lipids and plays an essential role in regulating energy metabolism [1]. NAFLD is a continuum of diseases that includes simple steatosis (lipid accumulation) and nonalcoholic steatohepatitis (NASH) and ultimately leads to cirrhosis, hepatocellular carcinoma (HCC), and end-stage liver failure developing in the absence of excessive alcohol intake. Simple steatosis is considered to have a benign hepatopathological prognosis. In contrast, NASH is characterized by the presence of steatosis, necroinflammation, and liver fibrosis and is associated with higher cardiovascular mortality that is largely caused by liver-related complications. NASH is a leading cause of liver transplantations [2]. The severity of NAFLD increases in parallel with other features of the metabolic syndrome, supporting the idea that NAFLD in obese individuals represents a hepatic manifestation of the metabolic syndrome. On the other hand, NAFLD has the potential to progress through the inflammatory phase of NASH to fibrosis, cirrhosis, and, in some cases, liver failure or HCC [3]. The disease etiology also involves significant redox and oxidative stress components [4–7].

### 1.2. Prevalence

The World Health Organization estimates that over one billion adults worldwide are overweight, at least 300 million of which are obese [8]. The percentage of obese individuals in the US increased from 12.0% in 1991 to 35.7% in 2010. In fact, currently, obesity is the second leading cause of preventable death in the US. Obesity represents a major public health challenge, and NAFLD represents a major complication of obesity. Because of the high prevalence of obesity, NAFLD and NASH have now reached alarming proportions, affecting 10–30% of the world's population. Despite of this, the Food and Drug Administration has not approved any specific treatment for either condition [9–11]. According to a recent study, 417,524 individuals in the US are living with NASH-associated cirrhosis, which represents a major complication of the disease, and approximately 4,104,871 individuals are living with NAFLD-associated advanced fibrosis [12]. Obesity and metabolic syndrome-related NAFLD are characterized by excess fat deposition in the liver, and this is associated with type 2 diabetes mellitus, hyperlipidemia, and insulin resistance [13–15].

### 1.3. NAFLD in Obese and Nonobese Individuals

The etiology of metabolism-related NAFLD in obese individuals is probably different from that of NAFLD diagnosed in nonobese individuals. The prevalence of NAFLD in nonobese individuals can reach 27% in the lean general population and is particularly common in Asian countries [16]. The pathophysiology of nonobese NAFLD is also possibly quite different from that of obese NAFLD. Genetic predisposition, a fructose- and cholesterol-rich diet, visceral adiposity, and dyslipidemia potentially contribute to the pathogenic process [16, 17]. Distinct causes of nonobese NAFLD, particularly in patients with nonmetabolic syndrome, remain unresolved, but could be connected to cholesterol and toxic bile acid levels in the liver and gastrointestinal (GI) tract. Lipotoxic effects can be mimicked by high-cholesterol diet (HCD), even in the absence of high fat content [18–20].

Categorizing the NAFLD severity is controversial because of contradicting epidemiological reports. NAFLD in the nonobese population has been increasingly reported, and the pathogenesis of nonobese NAFLD is poorly understood [21]. It was previously suggested that the long-term prognosis of nonobese NAFLD patients is worse than that of obese NAFLD patients, with a higher mortality rate among nonobese patients, even in those with a normal metabolic profile [22, 23]. In another report, metabolic syndrome parameters were recorded in nonobese NAFLD but with lesser magnitude relative to those noted in the obese individuals [24]. Obesity was identified as a major risk factor for the deterioration of NAFLD to fibrosis, as determined by a systematic search of Sookoian and Pirola (up to July 2017), which allowed a comparison of 493 nonobese patients and 2209 overweight or obese patients. The analysis revealed that fibrosis scores of overweight or obese-NAFLD patients were higher than those of nonobese NAFLD patients [25].

NAFLD can be detrimental to both obese and nonobese patients. Metabolic effects in nonobese NAFLD patients are less pronounced than the effects of direct liver damage resulting from an exposure of the liver to lipotoxic lipids, especially cholesterol and free fatty acids (FFA). Indeed, in animal models of NASH (methionine- and choline-deficient diet or atherogenic diet), loss of body weight, low levels of glucose, and depletion of the adipose tissue are observed when administered in the absence of high fat. On the other hand, liver damage and inflammation are much prominent in those models [18]. Therefore, NAFLD progression is not merely associated with excess caloric intake, and lipotoxicity could be the main factor that promotes the fatty liver disease progression, regardless of the metabolic impairment [26]. Lipotoxic effects could be critical in leading up to the nadir of liver function (in terms of clinical parameters) in both nonobese and obese NAFLD patients. Key landmarks in understanding the disease progression in terms of the lipotoxic effect of lipids are listed in Table 1.

## 2. Involvement of Lipids in Hypoxic Signaling: From Simple Steatosis to HCC

### 2.1. Hypoxic Signaling

Liver steatosis, inflammation fibrosis, and the formation of HCC are linked with redox signaling and hypoxic signaling via hypoxia-inducible factors (HIFs). HIFs are transcriptional regulators that control gene expression during hypoxia, enabling different cells to survive in the hypoxic environment and under stress conditions [38]. HIFs also regulate cell survival and cancer progression [39]. HIFs are members of the bHLH-PAS family of transcription factors and bind canonical DNA sequences, hypoxia response elements (HREs).

HIFs are heterodimers composed of *α* (HIF *α*) and *β* (ARNT/HIF *β*) subunits that activate the expression of hundreds of genes that encode proteins regulating cell metabolism, survival, mortality, basement membrane integrity, angiogenesis, vascular tone, hematopoiesis, and other functions [40]. The transcriptional activation and stabilization of HIF-1*α* increases when the local oxygen concentration is reduced. HIF-1*α* and HIF-2*α* undergo posttranslational modifications catalyzed by oxygen-dependent prolyl hydroxylases. These modifications typically stabilize HIF-1*α* in cells, proportionally enhancing HIF-1*α* activity. In hepatocyte cell lines, HIF-1*α* expression is regulated by stress-responsive deacetylase sirtuin 1 [41]. In contrast with the transient nature of HIF-1*α* activation and its involvement in the initial response to hypoxia, HIF-2*α* protein levels vary to a lesser extent and HIF-2*α* stabilization in the liver following its activation lasts longer than that of HIF-1*α* [42].

### 2.2. Hypoxia and Liver Diseases

Most chronic liver conditions are associated with hypoxic conditions linked to metabolic diseases, such as NAFLD. For example, obstructive sleep apnea syndrome (OSAS) and NAFLD are common conditions, frequently encountered in patients with metabolic disorders. OSAS has been associated with an increased risk of cardiovascular and metabolic complications. It was suggested recently that the chronic intermittent hypoxia during OSAS may also affect the occurrence and severity of NAFLD [43]. Chronic exposure of rats to hypoxic conditions resulted in increased activity of the transcription factors HIF-1*α*, AP-1, and nuclear factor (NF) *κ*B, which may be partially involved in hepatic responses to oxidative stress and liver injury under chronic hypoxia. Elevated expression of VEGF, ET-1, inducible nitric oxide synthase (iNOS), and endothelial NOS (eNOS) in response to chronic hypoxia were also reported [44].

NAFLD patients have increased risk to cholelithiasis [45]. Recently, using a NASH model of atherogenic diet supplementation in mice, the connection between hypoxic signaling, liver cholesterol accumulation, and gallstone formation was demonstrated. Protection against gallstone formation was demonstrated in iH-HIFKO mice (mice with specific HIF knockout in hepatocytes). Without HIF-1*α*, response to cholesterol lipid concentration was reduced compared with control mice, and bile flow increased due to hepatic expression of aquaporin 8 (AQP8) protein. In addition, liver tissues from patients with NAFLD with gallstones had increased levels of HIF-1*α*, HMOX1, and VEGFA mRNAs, compared with livers from patients with NAFLD without gallstones [46].

### 2.3. Liver Steatosis

The involvement of HIF-1*α* in liver steatosis has been linked to hypoxia-inducible protein 2 (HIG2), which is regulated by HIF-1*α*. The HIG2 protein is located at the hemimembrane of LDs and colocalizes with the LD proteins adipophilin and TIP47 [47–49]. HIG2 overexpression under normoxic conditions increases neutral lipid deposition in HeLa cells and stimulates cytokine expression [49]. HIG2 is detected in the atherosclerotic arteries and in patients with fatty liver disease, suggesting that this product of the ubiquitously inducible HIF-1*α* gene target may play an important functional role in disease progression and etiology associated with ectopic lipid accumulation [49]. Exposure of human cells to hypoxia reportedly causes accumulation of triglycerides and LD formation [50].

Another mechanism of HIF-1*α*-mediated lipid accumulation involves the induction of the lipin 1 gene, whose product is involved in triglyceride biosynthesis. HIF-1*α* reportedly binds a single distal HRE in the lipin 1 gene promoter, causing its activation under low-oxygen conditions [50]. Activation of the HIF-1*α* pathway by nitric oxide (NO) donors can also lead to lipid accumulation in hepatocytes. Treatment of the AML-12 mouse hepatocytes with the NO donor diethylenetriamine NONOate (DETA-NO) resulted in a dose- and time-dependent increase in lipid accumulation in these cells, as determined by Nile red fluorescence [51]. Further, exposure of the cells to 1 mM DETA-NO for 24 h resulted in elevated reactive oxygen species (ROS) production, mainly peroxides. NO induced HIF-1*α* expression, whereas treatment with the HIF-1*α* inhibitor YC-1 blocked lipid accumulation in these cells [51].

### 2.4. Liver Fibrosis

HIF-1*α* is also a major regulator of liver fibrosis [52–55]. In the context of progressive chronic liver disease under hypoxic conditions, activated myofibroblasts exhibit both proangiogenic and profibrogenic activities [56]. The product of the lysyl oxidase (LOX) gene, a hypoxia-responsive gene, catalyzes collagen crosslinking and is thought to be important in cancer metastasis and osteoarthritis. LOX is upregulated by both HIF-1*α* and HIF-2*α* [57]. In addition, LOX has been shown to significantly contribute to collagen stabilization during liver fibrosis [58].

**Figure 1:**
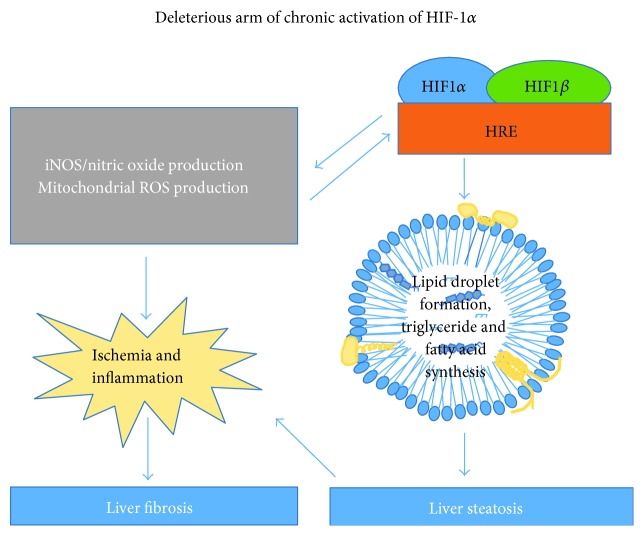
Consequences of chronic activation of the HIF-1*α*–iNOS axis, and its downstream involvement in lipid metabolism and fatty liver disease formation. HIF-1*α* stabilization can be induced by hypoxia or by exclusive NO or mitochondrial ROS production. HIF-1*α* stabilization promotes lipid synthesis and LD formation, both of which can aggravate liver steatosis. Chronic, but not transient, expression of HIF-1*α* and iNOS can induce inflammatory liver damage and fibrosis.

HIF proteins are potential target for treating chronic liver diseases [59]. Studies of a specific model of cholestasis revealed that chronic liver injury activates HIF-1*α* in macrophages, regulating the production of mediators of liver fibrosis. In fact, nuclear HIF-1*α* is present in macrophages, hepatocytes, and fibroblasts in the cholestatic liver disease, in the livers of patients with primary biliary cholangitis and primary sclerosing cholangitis [60]. Further, the levels of smooth muscle *α*-actin and type I collagen are lower in the liver of HIF-deficient mouse than those in a mouse with normal HIF levels, in a model of abstractive cholestasis of bile duct ligation [55]. These findings demonstrate that HIFs are important regulators of liver fibrosis [60] (Figure 1) and that their activation may be regulated by cholesterol accumulation in the liver.

### 2.5. HCC

The final stage of NAFLD is the formation of HCC. HIF-1*α* and HIF-2*α* were suggested to play pivotal roles in inducing HCC. These two proteins and NF-*κ*B have been shown to regulate genes involved in carcinogenesis and HCC progression. The von Hippel–Lindau (VHL) protein targets HIF-1/2*α* subunits for degradation and participates in modulating the activities of HIFs and NF-*κ*B. Recently, it was shown that pVHL overexpression synergizes with doxorubicin in the treatment of HCC [61]. Further, in an HCC model of injecting mouse HCC cells to the liver of immune competent mice, HIF-1*α* was shown to be associated with undifferentiation and accumulation of myeloid-derived suppressor cells (MDSCs), which exhibit immunosuppressive activities. In the report, it was suggested that HIF-1*α* may regulate tumor growth by regulating ectoenzyme, ectonucleoside triphosphate diphosphohydrolase 2 (ENTPD2), and extracellular levels of 5′-AMP to promote tumor growth through shaping the microenvironment of the HCC tumor in addition to direct impact. Thereby, MDSC accumulation enables cancer cells to escape immune surveillance and to become nonresponsive to immune suppression. Indeed, it was reported that hypoxia causes MDSC accumulation via the HIF-1*α* signaling pathway [62].

### 2.6. Glucose Metabolism and HCC

Under hypoxic conditions, cancer cells, including HCC cells, consume excessive levels of glucose as the major fuel source and produce high levels of lactate (via the Warburg effect, i.e., aerobic glycolysis) [63]. In HCC cells, the Warburg effect is controlled by HIF-1*α* [64]. In terms of clinical outcomes, the aggressiveness of HCC tumors may be attributed to the intensity of aerobic glycolysis. An elevated glycolysis enables tumors to survive under conditions of stress and to evade chemotherapy. Indeed, the activation of HIF-1*α*-dependent genes that regulate glycolysis is much higher in HCC with venous invasion than in HCC without venous invasion [65]. Another survival benefit is that in certain tumors (including HCC tumors), HIF-1*β*/ARNT expression is upregulated by HIF-1*α*, resulting in augmented HIF-1*α* signaling and better survival [66].

Taken together, these findings indicate that HIFs may serve as a novel and key therapeutic target for treating chronic liver metabolic diseases in human. HIF-1*α* inhibition could be relevant to the resolution of all NAFLD-related clinical parameters (including steatosis, chronic hepatitis, and fibrosis) and HCC, whereas its activation is relevant to the protection against ischemia/reperfusion- (I/R-) related injury and acute hepatitis [59]. Interestingly, dietary cholesterol can chronically activate the HIF-1*α* pathway even under normoxic conditions [20].

**Figure 2:**
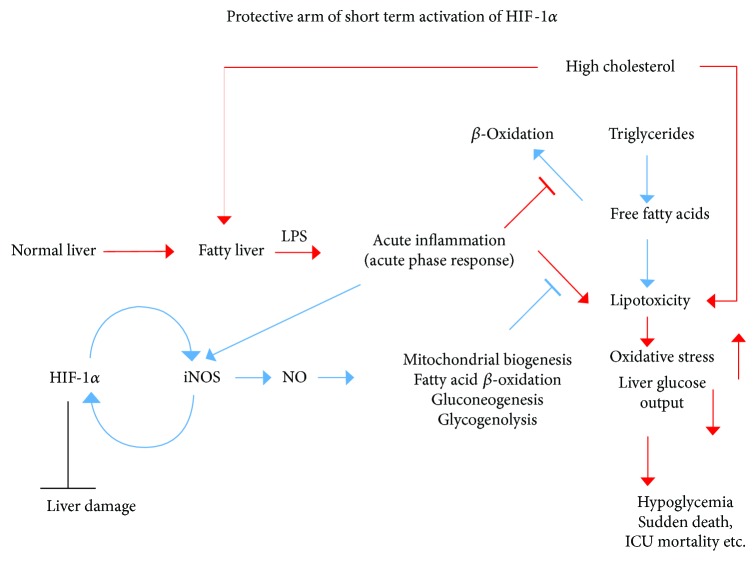
Consequences of acute activation of the HIF-1*α*–iNOS axis, and its downstream roles in lipid and glucose metabolism. Transient HIF-1*α* and iNOS activation in response to acute inflammatory signals can protect against metabolic collapse of the liver. This is especially relevant in the steatotic liver, allowing glucose and energy production under stress. ICU: intensive care unit.

The complex molecular function of HIF-1*α* is also relevant to its capacity to activate the expression of iNOS, one of the most important redox-signaling molecules, as well as oxidative and nitrosative stress-related enzymes [67]. iNOS can generate high levels of NO. Chronically produced NO can be deleterious while transiently produced NO can be protective in terms of correcting metabolic inflammatory stress (Figure 2).

Transient iNOS expression and activation of the HIF-1*α*–iNOS axis not only protects liver metabolism but also prevents the progression of liver damage [68]. Previously, it was postulated that NO may contribute to hepatotoxicity by mitochondrial activity inhibition, followed by reduced ATP synthesis, increased ROS production, and the inability to adapt to hypoxic stress [69]. It has been suggested that NO can block mitochondrial respiration and thereby prevent HIF-1*α* stabilization [70]. However, under normoxia, NO was shown to interact with the catalytic site of prolyl hydroxylase domain proteins and to promote HIF-1*α* stabilization [71]. Other observations imply that reduction of NO production by eNOS contributes to liver pathology by dysregulating the blood flow and oxygen delivery [72]. Furthermore, hepatocytes undergo necrosis and apoptosis after partial hepatectomy in iNOS-knockout mouse, indicating that NO production is essential for protecting hepatocytes from death after liver resection [73]. Knockout mouse models of iNOS or eNOS revealed that NO plays a crucial role in liver regeneration. Mei and Thevananther and Rai et al. reported impaired liver regeneration after partial hepatectomy in the eNOS- and iNOS-knockout mouse models, respectively [73, 74]. The role of NO in a treatment of partial hepatectomy was also demonstrated in animals supplemented with N(G)-nitro-L-arginine methyl ester (l-NAME), a NOS inhibitor. Impaired liver regeneration with simultaneously enhanced liver steatosis and reduced survival was observed in animals treated with l-NAME [75]. These data indicate that NO plays an antisteatotic role. Further, decreased eNOS expression precedes the formation of liver damage following intensive blood infusion of triglycerides in rat [76]. In summary, NO can be toxic or protective, depending on the liver microenvironment (Figure 2).

The specific roles of NO in NAFLD progression and liver fibrosis are ill defined. Marked fibrosis and inflammation are observed in the liver of iNOS-knockout mouse but not in wild-type (WT) mice after 48 weeks on a high-fat diet (HFD) [77]. However, following a short-term (6-week) supplementation of high cholesterol and cholic acid (the designated NASH model), chronic production of NO by iNOS induced liver fibrosis, HIF-1*α* stabilization, and DNA damage in WT mice [19].

Lipopolysaccharides (LPS) can promote liver inflammation and NASH [78]. However, conflicting reports fail to clarify the role of NO production in promoting this association. Although it has been suggested that NO is a mediator of organ dysfunction, some investigators have suggested that NO protects the liver and other organs. Previous studies from the author's laboratory demonstrated that iNOS-deficient mice with fatty liver induced by ethionine supplementation in choline-deficient diet, or a cholesterol/cholic acid-rich diet, are more sensitive to LPS treatment than WT mice are [68, 79]. It is known that fatty liver sensitivity to acute inflammation injury is much higher than that of normal liver. In a mouse model of fatty liver and endotoxemia, iNOS expression plays an important protective role [79].

Taken together, previous findings indicate that a feedback loop exists between HIF-1*α* and iNOS, protecting against cholesterol or LPS-induced metabolic collapse under stress (Figure 2). However, chronic activation of HIF-1*α* and iNOS can result in liver fibrosis and liver damage (Figure 1).

## 3. Cholesterol Toxicity and Metabolic Effects in NAFLD

NASH involves hepatic steatosis and necroinflammation. The transition towards hepatic inflammation represents a key step in disease pathogenesis because it promotes liver damage, culminating in hepatic fibrosis, cirrhosis, and liver cancer [3]. It is well known that phytosterol and dietary cholesterol absorption are tightly regulated in the GI tract. While the absorption efficiency of other types of lipids (especially triglycerides) is approximately 98%, the efficiency of cholesterol absorption is on average around 50% [80]. It is controlled by the Niemann–Pick C1-like 1 (NPC1L1), a polytopic transmembrane protein localized at the apical membrane of enterocytes and the canalicular membrane of hepatocytes, which functions as the gatekeeper for cholesterol absorption. NPC1L1 is a transporter that facilitates intestinal free cholesterol (FC) absorption. It also counterbalances hepatobiliary cholesterol excretion [81]. In addition to NPC1L1, two other transporters (ABCG5 and ABCG8) that potentiate plant sterol and cholesterol efflux back into the intestinal and biliary lumen for fecal excretion regulate decreased cholesterol uptake [82]. This unique control mechanism slows down the rate of absorption of FC to the circulation and the bodily tissues. This mechanism prevents atherosclerosis and protects the liver against cholesterol lipotoxicity [81].

**Figure 3:**
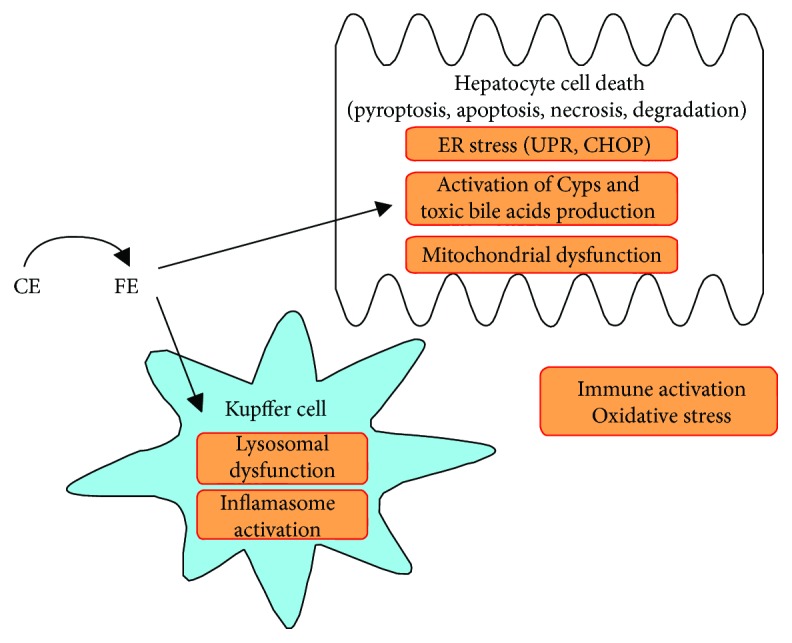
FC may directly or indirectly contribute to the development of hepatocyte lipotoxicity through different signaling pathways. Hydrolyzing cholesteryl ester (CE) to free cholesterol in the endosomes of Kupffer cells may lead to inflammation, oxidative stress, immune activation, and cell death. However, dietary FC can directly induce cell death in hepatocytes by different pathways, for example, activation of the Cyps pathway and induction of toxic bile acid production, mitochondrial dysfunction, and ER stress. CE: cholesteryl ester; Cyps: cytochrome P 450 enzymes; CHOP: C/EBP homologous protein; pyroptosis: type of cell death that involves caspase 1 activation and cell swelling [83]; UPR: unfolded protein response.

Emerging experimental and clinical data link altered hepatic cholesterol homeostasis and FC accumulation with NASH pathogenesis [83, 84]. When the experimental animals receive normal-fat diet supplemented with cholesterol (i.e., without HFD), the dietary cholesterol and liver cholesterol accumulation induce several NASH features with symptoms similar to those seen in nonobese human subjects with NASH. Such characteristics include a moderate loss of body weight, loss of adipose tissue mass, and little or no hyperinsulinemia [18]. Animals with steatohepatitis induced by methionine and choline deficiency and animals receiving an atherogenic (cholesterol + cholate) diet exhibit only minimal systemic insulin resistance. Insulin resistance is exacerbated by increasing the fat content (triglycerides) of the diets [30, 85]. This indicates that cholesterol is a nutritional factor critical for the development of NASH and that its lipotoxic activity is probably dissociated from the metabolic status of the patient [86, 87]. It has been known for a long time that cholesterol can induce apoptosis and plaque instability in macrophages by causing endoplasmic reticulum (ER) stress to promote thrombotic events [88]. The direct and indirect proapoptotic pathways associated with cholesterol in hepatocytes are shown in Figure 3. Although hepatic accumulation of triglycerides is linked to simple steatosis, it has become clear that cholesterol is involved in hepatic inflammation [89, 90].

The classic mechanism of cholesterol-induced NASH was initially proposed in 2006 [29]. It was suggested that mitochondrial FC loading is involved in precipitating NASH by changing the fluidity of the mitochondrial membranes, which led to the oxidation of mitochondrial glutathione, and sensitized hepatocytes to tumor necrosis factor (TNF) *α* and Fas-dependent death signaling via mitochondrial glutathione depletion [29]. Regarding the dietary effect on NASH, supplementation of leptin-deficient ob/ob obese mice on a HCD with high-fructose diet resulted in NASH development [91]. Rodents administered diets with high cholesterol content and cholic acid (atherogenic diets) developed steatohepatitis within 4–12 weeks [30].

Cholesterol can also alter the metabolic function and inflammatory status of the liver. Cholesterol in the form of modified plasma lipoproteins represents an important risk factor for the progression to hepatic inflammation in diet-induced NASH [89]. Further, hyperinsulinemia in conjunction with hepatic cholesterol accumulation activates the sterol regulatory element-binding protein 2 (SREBP-2) to upregulate a low-density lipoprotein receptor, which leads to reduced biotransformation of cholesterol to bile acids [92]. These events precipitate hepatocyte injury or apoptosis, macrophage recruitment, liver fibrosis, and progression from steatosis to NASH [92]. SREBP-2 accumulation in the liver was suggested to link between insulin resistance as a risk factor in NASH and necroinflammation. Its accumulation and activation in steatotic hepatocytes may be affected by multiple NASH-related factors including hyperinsulinemia, inflammatory cytokines, and miR dysregulation [92]. Such changes in metabolic conditions are significant contributors to liver FC lipotoxicity by increased cholesterol synthesis through the mevalonate pathway and due to increased cholesterol uptake (free and esterified) by the liver. Activation of SREBP-2 under conditions of insulin resistance can result in inhibition of mitochondrial *β*-oxidation leading to FFA accumulation. Therefore, insulin resistance can be connected to FFA toxicity.

In addition to direct lipotoxicity of FC, FFA, and other lipids, a mechanism for synergistic toxicity between cholesterol and FFA was suggested to be related to failure to activate the repression factor small heterodimer partner (SHP) upon farnesoid X receptor activation and was shown in obese NAFLD patients [93]. Altogether, this indicates that under NASH conditions there is probably an overaccumulation of cholesterol and bile acids in the liver. In animals, models of high-fat diets from plant source (with no cholesterol) induced insulin resistance rapidly without significant liver damage [94] indicating that access cholesterol in the liver is not pivotal for induction of insulin resistance but insulin resistance and metabolic syndrome could be important in precipitating lipotoxiciy in obesity.

In individuals with metabolic impairment, cholesterol may alter insulin metabolism, which is related to NASH. One study of metabolic syndrome-associated NASH in a rat model demonstrated a possible link between HCD and insulin signaling [95]. The study indicated that the effect of HCD on the development of hepatic insulin resistance is associated with the increased interaction between caveolin-1 and the liver insulin receptor. A mechanism was suggested whereby HCD alters caveolin-1 expression *in vivo*, which is accompanied by altered insulin receptor localization and activity [95]. Supplementation of rat diet with high cholesterol also induced insulin resistance, although elevated insulin receptor autophosphorylation (its activation) was observed in response to insulin [95]. Such contradicting effects of cholesterol on insulin signaling could be explained by a recent observation that both elevated and reduced plasma membrane cholesterol content affects insulin signaling in hepatocytes [96].

## 4. Signal Transduction Pathways for Dietary Cholesterol That Induce NASH

It is widely recognized that a Western-style diet increases the risk of NASH development and its subsequent progression to HCC [97]. However, the diet-induced changes in the signaling pathways relevant to these pathologies are not well understood. Several mechanisms have been proposed to explain the dramatic inducing effect of cholesterol on the progression of inflammation and apoptosis/necrosis of hepatocytes and nonparenchymal cells. Some of these mechanisms are related to redox signaling and oxidative stress. It has been suggested that the progression of NAFLD to steatohepatitis is underpinned by mitochondrial dysfunction, glutathione oxidation, and reduced mitochondrial membrane fluidity [29].

The paradoxical effect of cholesterol on hepatocytes and HCC, leading to cell death, was the subject of a recent review [98]. With respect to steatohepatitis, the effect of intracellular trafficking of cholesterol and its contribution to mitochondrial glutathione depletion in association with cell death was demonstrated. The loading of both dietary cholesterol and cholesterol arising from de novo synthesis probably affects mitochondrial glutathione carriers, resulting in mitochondrial glutathione depletion and sensitization of hepatocytes to inflammatory and apoptotic cytokines. The ER stress plays a limited role in the progression of NAFLD to NASH and was suggested not to be involved in cholesterol-induced NASH [29]. It was demonstrated that FC distribution in the ER and plasma membrane does not cause ER stress or alter inflammatory signaling [29]. However, the role of ER stress in cholesterol-induced NASH is limited to the steroidogenic acute regulatory protein-related lipid transfer domain protein StARD5, which may affect the ER in Kupffer cells. It was recently evidenced that ER stress induces the transcriptional upregulation of StARD1, facilitating mitochondrial cholesterol loading [98].

Interestingly, simple steatosis enhances the sensitivity of hepatocytes to hypoxic injury [99]. The author's research group demonstrated that reduced HIF-1*α* activation in steatotic hepatocytes compared to nonsteatotic hepatocytes is the reason for their increased vulnerability [99]. The capacity of steatotic cells to express HIF-1*α*-dependent genes responsible for the utilization of nutrients for energy production was also impaired. In contrast, overexpression of constitutively active HIF-1*α* significantly increased cellular viability and ATP and *GLUT1* mRNA levels in steatotic hepatocytes subjected to hypoxia. Further, in these cells, hypoxia led to the reduction of cellular and nuclear reduced glutathione levels and enhanced accumulation of 4-hydroxynonenal protein adducts. Hypoxia, in combination with hepatic steatosis, was also shown to promote oxidative stress, leading to NF-*κ*B inactivation and impaired HIF-1*α* induction, and thereby increasing cell susceptibility to hypoxic injury [99]. In contrast with steatotic hepatocytes loaded with TGs, hepatocyte treatment with cholesterol dramatically increases HIF-1*α* activation *in vitro* and *in vivo* and promotes molecular inflammatory response for cell survival, setting the stage for HCC induction [100]. An adaptive response of HCC cells to cholesterol is to protect mitochondrial glutathione levels from depletion via the 2-oxoglutarate carrier. The regulation of 2-oxoglutarate carrier expression was found to be HIF-dependent [100]. These findings indicate that the exposure of hepatocytes to cholesterol may lead to cell death (because of the effect of cholesterol on the mitochondria) or can activate a survival pathway specially in HCC.

In addition, the oxidative products of cholesterol oxysterols were suggested to contribute to liver injury and mitochondrial dysfunction. A synergistic interaction between FFA and oxysterols was suggested to impair mitochondrial function in NASH. Accumulation of specific nonenzymatic oxysterols and FFA induces mitochondrial damage and depletion of proteins of the respiratory chain complexes and mitochondrial biogenesis both *in vivo* and *in vitro* [101]. Targeted lipidomic analysis of a rat liver with steatohepatitis identified oxysterol triols (e.g., cholestane-3*β*,5*α*,6*β*-triol) that were associated with mitochondrial dysfunction and hepatocyte toxicity [102]. It was suggested that the hepatic accumulation of both fatty acids and toxic oxysterols, such as triols, leads to impaired mitochondrial function and biogenesis, contributing to liver pathology in NAFLD [102].

In addition to the classical mitochondrial damage hypothesis for the effect of cholesterol on hepatocytes, the following signaling pathways have been also suggested to mediate the damaging effect of cholesterol:

1. *Overactivation of the intestinal SREBP-2 transcription factor*: SREBP-2 activation was suggested to promote the progression of hepatic fibrosis associated with diet-induced NASH [103]. Mice specifically overexpressing SREBP-2 in the intestine exhibited greater inflammation and more severe fibrosis of the liver in response to HCD with HFD than their WT littermates. This demonstrates a novel link between the intestinal regulation of cholesterol metabolism and NASH pathogenesis [103].
2. *Cholesterol crystals in hepatocyte LDs and Kupffer cell activation*: In human and experimental NASH models, the mechanism underlying the enhanced proinflammatory effect of cholesterol was suggested to involve FC crystal formation in hepatocytes. This insight is important for understanding the progression of simple steatosis to NASH. Cholesterol crystals and crown-like structures that are formed in degraded hepatocytes were shown to interact with NLRP3 inflammasomes of Kupffer cells to induce inflammatory responses. This suggests that cholesterol can act as a damage-associated molecular pattern in the liver to promote activation of the NLRP3 inflammasome and other proinflammatory pathways [37, 104].
3. *Enhanced activation of Kupffer cells by LPS in the presence of toxic lipids*: Kupffer cells in the liver function in LPS clearance. Accumulation of lipids within hepatocytes and Kupffer cells can activate or suppress LPS activity and the bacterial load. Kupffer cells express high levels of class A scavenger receptors (SR-A). These receptors have affinity to modified lipoproteins, and LPS uptake may be overactivated due to the decreased capacity of steatotic hepatocytes to support LPS clearance thereby promoting NASH [105]. In addition, direct recognition of fatty acid moieties by Toll-like receptors (TLRs) is an important mechanism by which lipids regulate the inflammatory pathways and innate immunity in NAFLD/NASH patients [105].

FC metabolism may directly affect the proinflammatory activity of Kupffer cells. For example, in LDL receptor-deficient mice fed HFD, inflammation occurred only if the diet contained cholesterol [90]. The presence of foamy Kupffer cells suggests that scavenging of modified lipoproteins may induce inflammatory responses [90, 105]. In addition, the accumulation of cholesterol in the lysosomal fraction of Kupffer cells was suggested to facilitate liver inflammation [106].

(4) *FC promotes hepatic stellate cell (HSC) activation*: FC accumulation in HSCs induces liver fibrosis in NASH [19]. It has been suggested that FC activates HSCs by rendering them susceptible to transforming growth factor (TGF) *β* signaling [107]. The role of HSC activation by cholesterol was discussed in a recent review [7]. Indeed, FC activates HSCs in several animal models of NASH; for example, the inclusion of cholesterol in HFD in a methionine choline-deficient model or in models of cholestasis accelerates fibrosis [108]. The signaling mechanism suggested to explain that the activation of HSCs is associated with FC accumulation, which sensitizes the cells to TGF-*β* through TLR4 upregulation and downregulation of the TGF-*β* pseudoreceptor BAMBI (bone morphogenetic protein and activin membrane-bound inhibitor), leading to TGF-*β*-induced liver fibrosis [109].
(5) *Cholesterol-rich diet-induced protein kinase C β (*PKC*β*) *activation*: PKC*β* activation was suggested as a mechanism to prevent cholesterol accumulation in the liver and to protect against NASH development. Such a scenario indicates that PKC*β* represents an important mediator in the functional wiring of cholesterol metabolism. Indeed, the loss of PKC*β* activity induces tumorigenesis by modulating the stability of cell cycle-associated proteins [110]. Further, diets with high fat and high cholesterol content lead to NASH and HCC, and a systemic loss of PKC*β* promotes hepatic cholesterol accumulation in response to such diets. In addition, compared with nontumorous human liver specimens, reduced PKC*β* expression is observed in human HCC [110].
(6) *Chronic activation of hypoxia signaling pathways*: Plasma cholesterol levels correspond to decreased oxygen availability in the hepatic tissue, and the solubility and diffusion of oxygen are impaired in membranes with high cholesterol content [4]. The ability of cholesterol accumulated in the plasma membrane to ameliorate the diffusion of oxygen across membranes and to limit intracellular oxygen availability was demonstrated in several types of cells and in model membranes, as summarized in [4].

A mechanism whereby cholesterol loading of hepatocytes activates HIF-1*α* and induces chronic hypoxic responses was revealed *in vitro*, in isolated hepatocytes; *in vivo*, using an atherogenic diet; and in a bile duct ligation model of cholestasis [20, 55, 68]. HIF-1*α* activation was dependent on excessive production of reactive oxygen and nitrogen species by cholesterol–mitochondria interactions and iNOS activation [20]. This novel redox-signaling hypothesis can explain the transition from simple steatosis to NASH and liver fibrosis [19].

A possible physiological cause of HIF–NOS pathway activation is to correct and combat acute stress. It should be noted that gluconeogenesis and glycogenolysis are suppressed during acute inflammatory stress. It was suggested that this suppression of glucose production is associated with NO production [111]. However, using iNOS-deficient mouse, the author's research group has shown that NO generation actually supports glucose production in the liver [79]. Culturing hepatocytes with a combination of LPS, TNF-*α*, interleukin 1*β*, and interferon γ inhibits glucose generation by glycogen metabolism and prevents the repletion of glycogen in freshly cultured cells [112]. Further, a pyruvate tolerance test revealed that pretreatment of rats with LPS reduces hepatic gluconeogenesis [111]. The author's group also demonstrated that HIF-1*α* activation induces iNOS expression, supporting glucose production by NO signaling in the liver under inflammatory stress conditions [68]. Collectively, these findings indicate that such activation of the HIF-1*α*-NOS axis plays a role of a defense mechanism against acute inflammatory responses. This might be important under acute conditions, such as ischemic hepatitis. Ischemic hepatitis, described as “shock liver,” is characterized by a massive but transient increase in serum transaminase levels, usually associated with cardiac failure and hypoglycemia [113]. Another physiological situation that might result in glucose production failure is excessive consumption of alcohol during fasting, which can lead to severe hypoglycemia and sudden death [114] (Figure 2). Studies conducted using pharmacological approaches to stabilize HIFs have revealed the protective function of HIFs during I/R-induced liver injury [59]. Therefore, activation of HIF-1*α* and iNOS under liver stress conditions can protect against acute metabolic collapse. However, prolonged and chronic activation of the pathway is deleterious. Under chronic inflammatory conditions, such as NASH (Figure 1), this could lead to fibrosis and insulin resistance. This positive-feedback loop of HIF-1*α* activation leading to NO production to further stabilize HIF-1*α* levels (even under normoxia) may be continuously and strongly activated by cholesterol [20]. Indeed, a similar phenomenon of a positive-feedback loop between HIF-1*α* stabilization and the activation of iNOS expression was recently reported to operate during the inflammatory activation of macrophages [115]. In conclusion, the activation of HIFs likely occurs as an adaptive response to I/R-induced injury and acute metabolic stress. However, the consequences of prolonged activation of HIF and iNOS result in structural changes in the liver and damage that are relevant to NASH (Figure 1).

## 5. Clinical Relevance of Cholesterol and Cholesterol Level-Lowering Drugs in Liver Diseases

Understanding the risk factors and pathophysiology of NAFLD in nonobese and obese individuals is important. The hepatic cholesterol content is high, and hepatic cholesterol flux is robust [116, 117]. In addition, liver cholesterol levels were shown to be elevated in NASH patients [83, 118].

Despite its metabolic role in NASH development, the main function of cholesterol in NASH development is inducing liver damage via lipotoxicity (causing damage of hepatocytes and nonparenchymal liver cells). In that context, the effect of cholesterol accumulation in the liver would be most apparent in nonobese NAFLD patients, acting as a mechanism for the progression from simple steatosis to NASH and fibrosis in the absence of metabolic impairment. In addition, preventing hepatic absorption of dietary cholesterol by a drug treatment may constitute a good therapeutic strategy.

The NAFLD-promoting effect of cholesterol in obese individuals might be important from a metabolic point of view. Little clinical information is available regarding the specific effect of cholesterol on metabolism in NASH patients. Compared with normal liver, the fatty liver metabolism is altered in obese patients with NASH, as determined in the Kuopio Obesity Surgery Study [119]. The study involved 92 obese participants and confirmed that cholesteryl ester fatty acid composition was altered in NASH patients. Obese NASH patients with metabolic impairment would benefit from a treatment to improve the clinical lipoprotein profile.

The effect of ezetimibe (a drug that reduced cholesterol absorption and plasma cholesterol levels to treat NASH) was evaluated in a recent meta-analysis [120]. A significant reduction of liver enzyme activity in the serum, steatosis, and hepatocyte ballooning was observed. However, ezetimibe treatment did not ameliorate hepatic inflammation and fibrosis in patients with NAFLD and NASH [120]. Based on accumulated data, ezetimibe was suggested to affect only hepatocytes ballooning in NASH [121], but it is not recommended by the American and the European association of study of the liver.

Clinical trials indicate some positive effects of statins as cholesterol-lowering drugs for NASH treatment, as summarized by Pastori et al. [122]. One of the side effects of long-term statin treatment is elevated ALT levels in the blood; however, severe hepatic damage is rarely described. The relative safety of statin treatment was evidenced in 13 randomized, placebo-controlled trials in which statins were used for the treatment of hyperlipidemia and for secondary prevention of the cardiovascular disease. The observations supported the notion of safety of moderate doses of statins [122]. In patients exhibiting elevated liver enzyme levels and steatosis from the beginning of the trials, statin treatment does not exacerbate liver-related adverse effects [122]. Further, the frequency of such effects was low and did not differ from that among statin-untreated NAFLD patients. Moreover, a sustained 3-year treatment substantially ameliorated liver disease and improved blood liver enzyme levels in patients. Regarding the official recommendation of statin use for treating NAFLD and NASH, preliminary studies have shown that statins might improve the hepatic histology in patients [122]. Additional randomized controlled trials are required to assess the effect of statin administration on NAFLD activity score and liver fibrosis.

In another review [123], the authors suggested that statins are a safe NAFLD/NASH treatment and that their use is underappreciated. Three major prospective, randomized, controlled survival trials indicated the beneficial effect of statin use in NAFLD/NASH. These clinical trials demonstrated reduced cardiovascular disease (CVD) morbidity and mortality among statin-treated NAFLD/NASH patients compared to statin-treated patients without NASH. Statins reduced the number of CVD events in NAFLD/NASH CVD patients as compared to patients who were not receiving statin treatment [123]. Liver biopsy analyses revealed that NASH was resolved after a year of statin monotherapy, and liver enzymes, serum uric acid, and glucose returned to normal levels, and that statins exerted a protective effect against steatosis, steatohepatitis, and fibrosis [123]. Therefore, it is apparent that statin treatment is relatively safe, exerts a protective effect in human subjects with NAFLD/NASH, and might reduce cardiovascular disease-related morbidity and mortality. Altogether, these observations indicate that lowering endogenous cholesterol levels can significantly improve the symptoms and risk factors in obese NASH patients.

The clinical relevance of cholesterol in liver pathology may also be correlated with dietary patterns of NAFLD/NASH patients. Indeed, the diet of these patients appears to be rich in high saturated fat, cholesterol, and sweeteners [124]. In subjects with NAFLD, the consumption of high levels of fructose per day was associated with more extensive fibrosis [125]. In a study of 427 adults enrolled in the NASH Clinical Research, food questionnaires showed that fructose consumption classified into none, minimum to moderate (<7 servings/week), and daily (> or =7 servings/week) was associated with lower liver steatosis but higher fibrosis, increased hepatic inflammation, and hepatocyte ballooning [125]. This raises the possibility that fructose may promote the progression of simple steatosis to steatohepatitis [126]. Fructose might exert a proinflammatory effect because of its negative impact on the gut barrier and endotoxin leakage to the portal vein. It was recently demonstrated that fructose- and cholesterol-rich diets work synergistically to induce liver inflammation by affecting the gut barrier [127].

The cholesterol dose that results in liver toxicity in humans has not been established. However, it seems that both low-fat as well as low-carbohydrate diets are equally effective in the treatment of fatty liver disease and are implicated in some beneficial effects, for example, reducing ALT levels in the serum [126]. Regardless of the weight loss, therefore, restriction and modulation of dietary carbohydrates and reduction of the proinflammatory fat consumption (e.g., restriction of the total and saturated fat and cholesterol) may be beneficial to NASH patients, in addition to improving such metabolic parameters as insulin resistance and liver steatosis.

## 6. Conclusions

Current data regarding the effect of cholesterol loading in hepatocytes and nonparenchymal cells in the liver indicate that dietary cholesterol is a major nutrient that induces liver damage and lipotoxicity, both in obese and in nonobese individuals. Cholesterol might activate several redox, oxidative stress, and inflammatory signaling pathways to induce NAFLD progression. Its capacity to activate HIF-1*α* and iNOS is relevant to cholesterol-induced chronic liver diseases that are related to impaired lipid metabolism. Development of personal drug and dietary treatment strategies to ameliorate cholesterol lipotoxicity and to prevent sustained HIF-1*α* activation in NASH and NAFLD patients should be considered.

## Figures and Tables

**Table 1:** From triglycerides to toxic lipids: key landmarks representing progress in understanding lipotoxicity in NAFLD.

Year	Landmark	Significance	Ref.
1980	NAFLD characterized for the first time	Liver inflammation detected in Mobridge obesity patients	[27]
1998	The two-hit hypothesis	Inflammation occurs after fat (triglyceride) infiltration of the hepatocytes	[28]
2006	Role of free cholesterol (FC) in NASH described: mitochondrial dysfunction, oxidative damage, and proinflammatory effects	Activation of the immune system, inflammation, and cellular apoptosis, and hepatocyte necrosis	[29]
2007	FC and prooxidant effects recognized	Development of the atherogenic diet model for lipid-induced NASH	[30]
2007	Toxicity of free fatty acids described	Increased fibrosis and protective role of triglycerides	[31]
2008	Lipotoxicity of lysophosphatidylcholine determined	Death signals in hepatocytes induced by lipids	[32]
2010	The multiple-parallel hit hypothesis: NAFLD is a multifactorial disease	(a) Inflammation may precede steatosis or may be activated by failure of antilipotoxic protection (b) Other parallel hits derived from the gut and/or the adipose tissue may promote liver inflammation via multiple-organ crosstalk (c) Endoplasmic reticulum (ER) stress and its effect related to signaling networks for steatosis	[33]
2012	Lipids activate NLR family pyrin domain-containing 3 (NLRP3) inflammasomes.	Hepatic long-chain fatty acid composition, a novel determinant in inflammatory response and NASH development	[34]
2012–2014	Ceramide lipotoxicity recognized	Ceramide accumulation and altered acylation pattern in the liver are connected to hepatic steatosis, elevated plasma free fatty acid levels, insulin resistance, and lipotoxicity: these are all noted in NASH	[35, 36]
2017	Cholesterol crystallization within hepatocyte lipid droplets (LDs) observed	Activation of macrophages causes upregulation of tumor necrosis factor (TNF) *α*, NLRP3, and interleukin 1*β*. Cholesterol crystals formed on the LD membrane of degrading hepatocytes facilitate inflammatory activation of Kupffer cells	[37]

## Abbreviations

DETA-NO: Diethylenetriamine-NONOate
eNOS: Endothelial nitric oxide synthase
FC: Free cholesterol
FFA: Free fatty acids
HCC: Hepatocellular carcinoma
HCD: High-cholesterol diet
HFD: High-fat diet
HIF-1*α*: Hypoxia-inducible factor 1*α*
HIG2: Hypoxia-inducible protein 2
HRE: Hypoxia-response element
HSC: Hepatic stellate cell
I/R: Ischemia/reperfusion
iNOS: Inducible nitric oxide synthase
LD: Lipid droplet
LOX: Lysyl oxidase
LPS: Lipopolysaccharide
MDSC: Myeloid-derived suppressor cell
NAFLD: Nonalcoholic fatty liver disease
NASH: Nonalcoholic steatohepatitis
NF-*κ*B: Nuclear factor-*κ*B
NO: Nitric oxide
NPC1L1: Niemann–Pick C1-like 1
OSAS: Obstructive sleep apnea syndrome
ROS: Reactive oxygen species
TGF-*β*: Transforming growth factor *β*
TLR: Toll-like receptor
TNF-*α*: Tumor necrosis factor *α*
VHL: von Hippel–Lindau
WT: Wild-type.
